# Antibiotic Resistance during COVID-19: A Systematic Review

**DOI:** 10.3390/ijerph191911931

**Published:** 2022-09-21

**Authors:** Hadi Jaber Al Sulayyim, Rohani Ismail, Abdullah Al Hamid, Noraini Abdul Ghafar

**Affiliations:** 1 Interdisciplinary Health Unit, School of Health Science, Universiti Sains Malaysia (Health Campus), Kubang Kerian 11800, Kelantan, Malaysia; 2 Saudi Ministry of Health, Najran 11134, Saudi Arabia; 3 Biomedicine Program, School of Health Science, Universiti Sains Malaysia (Health Campus), Kubang Kerian 11800, Kelantan, Malaysia

**Keywords:** antibiotic resistance, common antibiotic-resistant bacteria, COVID-19, SARS-2

## Abstract

One of the public health issues faced worldwide is antibiotic resistance (AR). During the novel coronavirus (COVID-19) pandemic, AR has increased. Since some studies have stated AR has increased during the COVID-19 pandemic, and others have stated otherwise, this study aimed to explore this impact. Seven databases—PubMed, MEDLINE, EMBASE, Scopus, Cochrane, Web of Science, and CINAHL—were searched using related keywords to identify studies relevant to AR during COVID-19 published from December 2019 to May 2022, according to PRISMA guidelines. Twenty-three studies were included in this review, and the evidence showed that AR has increased during the COVID-19 pandemic. The most commonly reported resistant Gram-negative bacteria was *Acinetobacter* *baumannii*, followed by *Klebsiella pneumonia*, *Escherichia coli*, and *Pseudomonas aeruginosa*. *A. baumannii* and *K. pneumonia* were highly resistant to tested antibiotics compared with *E. coli* and *P. aeruginosa*. Moreover, *K. pneumonia* showed high resistance to colistin. Commonly reported Gram-positive bacteria were *Staphylococcus aureus* and *Enterococcus faecium*. The resistance of *E. faecium* to ampicillin, erythromycin, and Ciprofloxacin was high. Self-antibiotic medication, empirical antibiotic administration, and antibiotics prescribed by general practitioners were the risk factors of high levels of AR during COVID-19. Antibiotics’ prescription should be strictly implemented, relying on the Antimicrobial Stewardship Program (ASP) and guidelines from the World Health Organization (WHO) or Ministry of Health (MOH).

## 1. Introduction

On 11 March 2020, the WHO announced the COVID-19 pandemic [1]. The disease known as COVID-19 or SARS-2 spread rapidly from Wuhan City, China, to the rest of the globe [2]. As of early July 2022, oughly 547,901,157 COVID-19 cases and 6,339,899 deaths have been officially reported [3].

During the COVID-19 pandemic, there were improper uses of antibiotics either in healthcare institutions or in communities, which in turn played a role in the increase in AR [4,5,6]. It has been documented that about 72% of COVID-19-admitted patients were treated with antimicrobials, whereas solely 8% of these patients had bacterial or fungal co-infection [4]. Additionally, different antibiotics have been explored or suggested to cure COVID-19 patients, e.g., azithromycin [4,5]. Both the worry and the improper use of antibiotics directly impact access to antibiotics without a prescription, particularly low- and middle-income countries that have a weak system of antibiotic control. In this correlation, Zavala-Flores E et al., 2020, reported that nearly 69% of COVID-19 patients stated that they had used antibiotics (namely, ceftriaxone and azithromycin) before being admitted to the hospital [6].

Furthermore, during the COVID-19 pandemic, there was a huge increase in the use of biocides universally. These biocides probably encouraged more indirect pressure leading to AR [4]. Since early 2020, this situation has expanded globally and might have supported the evolution of extremely resistant microorganisms, which might have played a critical role in worsening the status of some patients, especially those who were admitted to intensive care units (ICUs). It has been reported that there were some deadly co-infections caused by pan-resistant microorganisms among COVID-19 patients. *S. aureus* and *A.* *baumannii* were the major ones that were resistant to extended-spectrum antibiotics, which were mostly used to cure life-threating diseases caused by bacterial infections [7].

Findings from a review stated that despite the bacterial infections associated with COVID-19, patients were less affected than in the influenza pandemic. COVID-19 patients were affected by common types of bacterial co-infection. These included *S. aureus*, *Streptococcus pneumoniae*, *Klebsiella* spp., *Mycoplasma pneumonia*, *Legionella pneumophila*, and *Haemophilus Sp.*, *Mycobacterium tuberclosis* as a co-infection among COVID-19 patients. The study, however, reported that the rates of secondary bacterial co-infection were high among COVID-19 patients admitted to ICU, which could be due to hospital-acquired AR bacteria. Consequently, the study recommended urgently revising the empirical broad-spectrum antibiotics prescribed to COVID-19 patients and considering the importance guidelines of ASP [8].

COVID-19 patients who were admitted to ICU mostly required intubation and were at risk of ventilator-associated pneumonia, especially Gram-negative bacteria (*P. aeruginosa*, *Acinetobacter Sp.*, and *K. pneumoniae*) and Gram-positive bacteria, (*S. aureus*). A study targeting five ICUs in Britain revealed that the prevalent bacteria among COVID-19 patients were *Klebsiella aerogenes* and *K. pneumonia* [9], whereas excessive levels of non-fermenters were found in one hospital in France [10]. COVID-19 patients on ventilators often received courses of multiple antibiotics. ASP guidelines were unfortunately overrun during the peak of COVID-19 as the capacities of ICUs increased [11]. In Spain, it was reported that the use of antibiotic increased [12], and as the pressure of COVID-19 increased, the resistance may have increased accordingly.

Another study has reported that the occurrence of multidrug-resistant organisms (MDROs) has increased in the era of COVID-19 compared with three years before the pandemic, and there was a high incidence of extended spectrum beta-lactamase (ESBL) *K. pneumonia* [13]. Furthermore, recent reports found that AR during the COVID-19 pandemic was higher than in previous periods [14,15,16]. Since there are some studies that have reported that AR increased during COVID-19 and others that have stated otherwise, the aim of this review was to explore the impact of COVID-19 on AR. The specific objectives were to identify the pattern of reported AR during the COVID-19 pandemic, to determine the nature of reported AR during COVID-19, and to report the encountered risk factors of AR during COVID-19.

## 2. Materials and Methods

**Figure 1:**
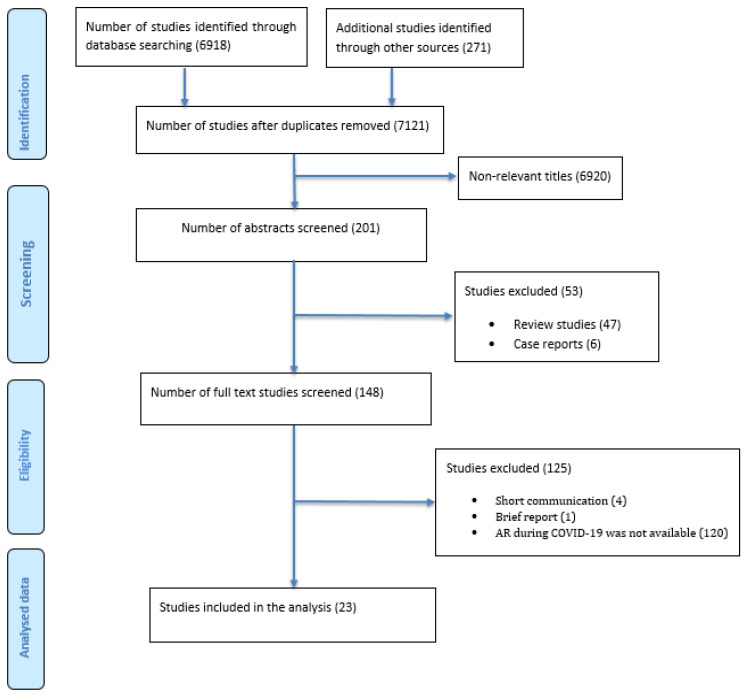
PRISMA flowchart of the systematic search.

Preferred Reporting Items for Systematic Reviews and Meta-Analyses (PRISMA) guidelines were followed for reporting in this systematic review (Figure 1) [17]. PRISMA is a set of evidence-based items to report systematic reviews and meta-analyses. It concentrates on reporting revisions which assess the impacts of interventions. It could be used for systematic reviewing without assessing the interventions such as the evaluation of the cause or diagnosis, etc. [17]. The protocol of this systematic review was registered on the PROSPERO database (CRD42022326361).

### 2.1. Inclusion Criteria

Studies were included based on the following criteria:

1. Articles should be original studies.
2. Studies should report data on at least these two variables: antibiotic resistance, and COVID-19.
3. Studies should be written in English or at least their abstract should be in English.
4. Studies should be published between 2019 (since announcing COVID-19 in the country where the included study conducted) and May 2022.

### 2.2. Exclusion Criteria

Studies were excluded if they were a case report, letter to the editor, conference articles, commentary, systematic review, or viewpoint. Studies were also excluded if they were written in a non-English language or reported AR in non-human populations.

### 2.3. Search Strategy

An electronic search was employed to find the published articles from December 2019 to 20 May 2022, which reported antibiotic resistance during COVID-19 through the following databases: PubMed, MEDLINE, EMBASE, Scopus, Cochrane, Web of Science, and CINAHL. The medRxiv database was also searched to ensure a comprehensive search for unpublished studies.

We employed the following search terms: ‘Antibiotic’, ‘resistance’, ‘COVID-19′. In addition, Boolean operators (OR/AND) and asterisk (*) were used to find available related evidence as follows: “Anti-Bacterial Agents” OR “Antibiotic*” OR “Antimicrobial*” OR “Anti-bacterial*” OR “Antibacterial agent*” OR “Bacteriocide*” OR “Bacteriocidal Agent*” AND “Drug Resistance” OR “Resistance OR Resistant*” OR “Antibacterial drug resistance*” OR “Antibiotic resistance*” OR “Antimicrobial resistance*” OR “Susceptible*” OR “Antibiotic Susceptibility” OR “Antimicrobial Susceptibility” AND “COVID-19” OR “COVID19” OR “Severe Acute Respiratory Syndrome Coronavirus 2” OR “SARS CoV 2 Infection” OR “SARS-CoV-2 Infections” OR “2019-nCoV Diseases“ OR “2019 Novel Coronavirus Disease” OR “COVID-19 Pandemic” OR “COVID 19 Pandemic” OR “Coronavirus Disease 19” OR “Coronavirus Disease-19”.

### 2.4. Study Selection and Data Extraction

The initial screening for the title and abstract was performed by A.H. and A.A., and the full text screening for the eligible studies was performed by all authors. Data extraction was carried out by all authors, using a detailed extraction sheet including the following data: first author, country, year, setting, study design, duration, sample size, age, antibiotic-resistance-related data, and causative bacteria. Disagreement between authors was resolved by a joint discussion.

### 2.5. Quality Assessment

The quality assessment of the included studies was assessed based on the Joanna Briggs Institute critical appraisal tool [18]. The tool has 8 items to assess cross-sectional studies and 11 items to assess cohort studies. Each cross-sectional study was scored from 0 to 8, and the cohort study was scored from 0 to 11. Subsequently, the quality of the included studies ranked as high (for score ≥70%), medium (for score 50–69%) and low (for score <50%) [19]. All authors performed the assessment, and the issues encountered during the assessment were resolved by discussion among the authors.

### 2.6. Data Analysis

Data analysis was carried out using Microsoft Excel 2016. Median and IQR were used to present the resistance of each bacterium against various antibiotics. In this systematic review, the resistance of bacteria to the tested antibiotics, which was reported in more than three studies, was combined to identify the median and IQR of AR. In addition, an analysis for each study was performed narratively for the relevant data (AR findings, nature of AR bacteria, and potential risk factors).

## 3. Results

### 3.1. Study Characteristics

The search strategy yielded 7189 studies: PubMed (875), Scopus (1367), Medline (217), Embase (2086), Web of Science (2325), CINAHL (24), and Cochrane (24), and an additional 271 studies were retrieved from medRxiv (Figure 1). After removing the duplicates, 7121 studies remained for title and abstract screening. In total, 148 studies were eligible for screening the full text, of which 125 did not meet the inclusion criteria for the following reasons: short communication (4), brief report (1), and no relevant AR data (120). Thus, 23 studies met the inclusion criteria and were included in this review.

The summary of the characteristics and findings of the 23 included studies are presented in Table 1. The majority of the studies were from Iran (4) and India (4), followed by 2 studies from each the following countries: China, Italy, Turkey, and Saudi Arabia. Only 1 study was from each of the following countries: New York, Serbia, Egypt, Pakistan, Indonesia, Switzerland, and Greece. The majority of the studies employed a retrospective study design (10), followed by a retrospective observational (6), retrospective record review (3), and cross-sectional study (2). One study was a retrospective cohort study, and another was a retrospective follow-up study. Out of the 23 studies, 17 studies reported that AR emerged from ICUs, whereas only 6 studies reported some patient care areas in addition to ICUs (Table 1).

The majority of the studies (17) reported their number of samples as ranging between 13 and 856 patients, whereas only one study had 3532 patients. In total, 5 studies reported their samples as isolates ranging from 168 to 286 isolates, and only 1 study had 17,837 isolates. Only 1 study included 7309 samples pre-pandemic and 4968 samples during the pandemic phase in 2020, such as blood and urine samples. The majority (8) of studies reported the patient’ ages as a median ranging from 56 to 67 years, whereas 6 studies reported ages as a mean ranging from 46 to 71 years, 3 studies classified ages as groups, and only 1 study presented the age as a range from 40 to 83 (Table 1).

### 3.2. Antibiotic Resistance Findings during COVID-19

The majority of the studies has reported high AR during the COVID-19 pandemic [7,20,21,22,23,24,25,26,27,28,29,30,31,32,33]. One study reported the highly increased the resistance of *A. baumannii* to all tested antibiotics except colistin [7]. The rate of AR was generally high, where carbapenem-resistant *A. baumannii* and carbapenem-resistant *K. pneumoniae* accounted for 91.7 and 76.6%, respectively. *S. aureus* and Coagulase-negative *staphylococci* were resistant to methicillin (100%). Resistance of ESBL producing *E. coli* was 75% [20,23]. Different tested isolates against meropenem and imipenem showed high resistance, ranging from 50% to 100% [21,23,28,29,34]. MDRO and carbapenem-resistant *K. pneumonia*, *A. baumannii*, and *Pseudomonas* spp. were high in China, where they exceeded 92% (Table 1) [22].

Eight studies found a high resistance of *A. baumannii* to the tested antibiotics, ranging from 90 to 100% [7,20,22,27,30,31,34,35]. The resistance of *K. pneumonia* was reported in seven studies, indicating high resistance (94%–100%) [21,22,27,30,31,34,35]. One patient with *K. pneumoniae* had a failure of treatment with ceftazidime/avibactam due to the development of resistance [21]. One study found that most patients had extensive drug resistance (XDR) [28], and another study highlighted *K. pneumonia* as the most frequent pan-drug-resistant (PDR) bacteria (Table 1) [35].

Three studies were interested in identifying the rate of AR during or after COVID-19 compared with the era before the pandemic [23,33,36]. One study found that AR to imipenem, meropenem, and ciprofloxacin was significantly higher than the era before COVID-19 [23]. The prevalence of the resistance of *S. aureus* to oxacilin and *Conrynebacterium striatum* to vancomycin and linezolid during COVID-19 was higher than during the pre-pandemic era (Table 1) [33]. On the other hand, the rate of ESBL-producing Enterobacterales (MDROs bacteria) was similar in the era before and during COVID-19 [32]. The AR rates were similar before and during the COVID-19 pandemic (Table 1) [37].

### 3.3. Nature of AR during COVID-19

In total, four Gram-negative bacteria and two Gram-positive bacteria were commonly reported. Of the 23 included studies, 16 studies reported *A. baumannii* as one of the most common resistant bacteria, followed by *K. pneumonia* (15 studies), *E. coli* (10 studies), and *P. aeruginosa* (9 studies). However, among Gram-positive bacteria, *S. aureus* was mentioned in 3 studies as one of the most frequently resistant bacteria, followed by *E. faecalis* in 1 study, and *E. faecium* in another study (Table 1).

### 3.4. Pattern of Resistant Bacteria to Tested Antibiotics during the COVID-19 Pandemic

Details of resistant Gram-negative bacteria to tested antibiotics during COVID-19 are integrated in Table 2 from 12 studies [20,23,25,28,30,31,34,36,37,38,39,40]. The resistance of *A. baumannii* was high to levofloxacin Median (M) 97.05% (IQR 91.92–100%), gentamicin M 95.7% (IQR 74.2–97.1%), cefepime M 94.4% (IQR 93–100%), and piperacillin/tazobactam M 93.7% (IQR 66.9–100%). The resistance of *A. baumannii* was also high for the following antibiotics: ceftazidime, meropenem, imipenem, and ciprofloxacin with the same M, 91.2%, with the IQR ranging between 50 and 100%. The resistance to amikacin and ceftriaxone was also increased: M 84.6% (IQR 56.3–92.95%) and M 76.2% (IQR 54.75–95.55%), respectively. However, the resistance to colistin and tigecycline was low: M 2.5% (IQR 0–19.62%) and M 9.5% (IQR 8.8–33.3%), respectively. There were high levels of AR observed in *E. coli* to ampicillin M 87.5% (IQR 85.25–93.75%), amoxicillin clavulanate M 85.5% (IQR 49–92.75%), levofloxacin M 75% (IQR 56.85–87.5%), ceftriaxone M 73% (IRQ 49.25–93.75%), ciprofloxacin M 71% (IQR 28.2–76%), and cefuroxime M 65.5% (IQR 55.75–77.62%). Notably, very low levels of AR were observed in *E. coli* to cefepime M 0% (IQR 0–25%), colistin M 0% (IQR 0–7.15%), and tigecycline M 0% (IQR 0–7.15%).

Ampicillin, cefazolin, and ceftazidime resistance in *K. pneumonia* was M 100% (IQR 90.5–100%), M 93% (IQR 78–95.5%), and M 93.5% (IQR 83.7–97.9%), respectively. The median resistance of *K. pneumonia* to trimethoprim/sulfamethoxazole was 73.5% (32–74%). Although the AR level in *P. aeruginosa* was low, the resistance to ceftriaxone was M 75% (IQR 43.75–87.5%) (Table 2).

The susceptibility of *S. aureus* and *E. faecium* to seven antibiotics was identified in eight studies [20,24,28,30,34,36,38,39]. *E. faecium* showed high resistance to erythromycin M 90.9% (IQR 78.45–95.45%), ciprofloxacin M 81.8% (IQR 77–100%), and ampicillin M 81.8% (IQR 52.4–90.9%), whereas the resistance in *S. aureus* was M 48.5% (IQR 25.5–63.75%) and M 33.3% (IQR 16.65–50.9%) to oxacillin and clindamycin, respectively (Table 3).

### 3.5. Potential Risk Factors

The risk factors of AR during COVID-19 were explored in only three studies. Self-antibiotic medication and antibiotics prescribed by general practitioners were significant risk factors for high levels of AR among the COVID-19 group compared with the non-COVID-19 group [24]. Another study reported that the administration of empirical antibiotics prior to ICU admission resulted in a high prevalence of MDRO [26]. In a study conducted in Iran, it was observed that 100% of patients who had MDR superinfection were imposed to empirical antibiotics, namely, meropenem and levofloxacin, with a median of duration of 12 and 9 days, respectively [28].

## 4. Discussion

In this systematic review, we examined the findings of 23 included studies that reported AR during COVID-19, and in three of them the reported risk factors were summarized. AR levels during COVID-19 were high, and the most commonly reported antibiotic-resistant Gram-negative bacteria were *A. baumannii*, *K. pneumonia*. Despite all Gram-negative bacteria in this study showing no resistance to colistin, *K. pneumonia* was high. Commonly reported Gram-positive bacteria were *S. aureus* and *E. faecium*, and a high resistance of *E. faecium* to ampicillin, erythromycin, and ciprofloxacin was observed. Self-antibiotic medication, empirical antibiotic administration, and antibiotics prescribed by general practitioners were the risk factors for high levels of AR during COVID-19.

Regarding the most commonly reported AR bacteria, a systematic review in 2019 reported *E. coli* as a common AR bacteria [41]. Another study reported the common Gram-negative bacteria as follows: *P. aeruginosa*, *Klebsiella* spp., *A. baumannii*, *E. coli*. Coagulase-negative *Staphylococcus*, *Enterococcus* spp., and *S. aureus* were the common Gram-positive bacteria [42]. Additionally, *E. coli* was a previously common resistant bacteria, followed by *S. aureus* [43,44]. Our findings are congruent with previous studies; however, among Gram-negative bacteria, *A. baumannii* and *K. pneumonia* were the most commonly reported ones.

In the present review, the resistance of *A. baumannii* to amikacin, cefepime, ceftazidime, gentamicin, meropenem, imipenem, ciprofloxacin, and piperacillin/tazobactam was higher than previously published studies before COVID-19. In a study carried out to report AR over five years before COVID-19, findings reported that *A. baumannii* was resistant to amikacin (49%), cefepime (78.6%), ceftazidime (73.8%), ciprofloxacin (46.7%), and piperacillin/tazobactam (62.2%). However, the resistance to meropenem was similar to the levels during COVID-19, and for imipenem this was 82.7%, which was still not higher than the levels observed during COVID-19 in the present study [42]. Another study conducted in 2019 mentioned that *A. baumannii* isolates were 66% resistant to the tested antibiotics, except colistin, which showed no resistance [45]. The WHO issued a report (2014–2019) illustrating the pattern of carbapenem resistance in *A. baumannii*, which was much lower than in the present study.

This review found that the resistance of *E. coli* isolates to amoxicillin clavulanate, cefuroxime, ceftriaxone, levofloxacin, and ciprofloxacin was increased during COVID-19. In comparison, the resistance of *E. coli* before COVID-19 to ciprofloxacin and levofloxacin was 46% and 43%, respectively [42]. In China, in a study conducted to monitor AR for about 12 years until 2019, the resistance of *E. coli* to piperacillin/tazobactam did not exceed 8%, and resistance to ciprofloxacin did not exceed 60% [46]. Similarly, the AR of *E. coli* before COVID-19 to piperacillin/tazobactam had a median of 12%, as well as ciprofloxacin (65%), ceftriaxone (59%), and levofloxacin (62%) [47].

In 2018, a systematic review reported that the resistance of *K. pneumonia* isolates to tested antibiotics and the resistance in amikacin was 37%, ceftazidime was 82%, ceftriaxone was 78, levofloxacin was 54%, meropenem was 7.7%, imipenem was 0%, ciprofloxacin was 67%, and nitrofurantoin was 39% [47]. A retrospective study of AR patterns from 2013 to 2018 reported that the resistance in *K. pneumonia* to amikacin was 34%, cefuroxime was 71%, ceftazidime was 67%, levofloxacin was 12.8%, imipenem was 18%, and ciprofloxacin was 21% [42]. Additionally, a systematic review carried out in 2019 reporting AR in *K. pneumonia* reported the following: amikacin (40.8%), ceftazidime (75.7%), ciprpfoxacin (59.8%), colistin (2.9%), cefotaxime (79.2%), cefepime (72.6%), meropenem (62.7%), imipenem (65.6%), levofoxacin (54.1)%, and trimethoprim sulfamethoxazole (58.2%) [48]. The findings of AR in the current review were much higher than in previous studies in 2019 and in previous years.

A study concerning AR in *P. aeruginosa* including 18 countries worldwide showed a low resistance to amikacin, gentamicin, ceftazidime, imipenem, ciprofloxacin, and levofloxacin [49]. A systematic review reported the resistance to imipenem in 2006, which was 42% and dropped gradually to 23% in 2017; moreover, the resistance to ciprofloxacin ranged from 32% to 14% over 11 years prior to COVID-19 [46]. The percentage of AR to carbapenem-resistant bacteria between 2014 and 2019 was 5% [43]. The findings of our review were not in line with previously published articles; moreover, the resistance to imipenem and ciprofloxacin was almost two times higher.

Colistin is an important antibiotic for various types of Gram-negative bacteria, and is the last resort for physicians to treat bacterial infections involving E. coli [50]. Previous studies reported very low resistance to colistin [47,48]. Notably, in our review, the resistance of *K. pneumonia* to colistin increased during COVID-19, with a median of 21.1% (IQR 12.42–69.82%).

Regarding the resistant Gram-positive bacteria, in the period from 2015 to 2019, the resistance of *S. aureus* to clindamycin lay between 17 and 15%, and the pattern of *Enterococcus* species resistance to ampicillin was 5–35%, erythromycin was 65–85%, ciprofloxacin was 60–80%, vancomycin was 10–50%, and tetracycline was 40–80% [44]. In other systematic reviews, *S. aureus* resistance to clindamycin was 11.7% and oxacillin was 34.5–46% [41,47]. In contrast, in the present review, the resistance of *E. faecium to* ampicillin, erythromycin, and ciprofloxacin was higher during COVID-19 than before. The resistance of *S. aureus* to clindamycin was 33.3% and oxacillin was 48.5%, which was still higher than before COVID-19.

Regarding the risk factors, it is important to note that sometimes antibiotics are self-administered by individuals or prescribed by physicians to avoid bacterial colonization, even with no specific bacterial infection or laboratory-based confirmation. However, antibiotic treatment should be used based on accurate diagnosis [51]. About 72% of COVID-19-admitted patients in hospitals were treated with antimicrobials, whereas about 8% of these patients had bacterial or fungal co-infection [4]. Nearly 69% of COVID-19 patients stated that they had used antibiotics (namely ceftriaxone and azithromycin) before being admitted to the hospital [6]. In the present review, self-antibiotic medication, antibiotics prescribed by general practitioners, and empirical antibiotics prior to ICU admission were the reported risk factors of AR during COVID-19. A recent systematic review assessing the risk factors of AR from 2013 to 2019 reported a similar risk factor, which was current or previous exposure to antibiotics; however, other factors included sociodemographics and admission to hospital [52].

### Strengths and Limitations

This review has many strengths as the first systematic review addressing the impact of COVID-19 on AR and the relevant risk factors, based on the analysis of the retrieved evidence from thirteen countries worldwide. Additionally, the data from recent studies conducted during COVID-19 were included in this review, which in turn provided up-to-date data on the impact of the COVID-19 pandemic on AR, as well as relevant risk factors.

On the other hand, there are some limitations. First, a potential limitation was our approach of incorporating AR from various groups of patients from various countries to measure the resistance percentages of bacteria to different antibiotics. In this approach, high resistance in various healthcare settings may have balanced out. Nevertheless, given the observed patterns, it is highly probable that the accuracy of the gathered data was enough to display the overall situation. Second, the extracted and related AR data were measured by different laboratory procedures. However, although the guidelines of the Clinical Laboratory Standards Institute and standard disc diffusion were mostly employed in the studies, it is believed that the validity of the outcomes would not be affected. Third, the global generalization of the study findings was another potential limitation. Although there were 23 retrieved studies across 13 countries worldwide, the studies were from different settings, populations, and healthcare systems, which provided an overall view regarding the impact of COVID-19 on AR and related risk factors.

## 5. Conclusions

AR during COVID-19 was high, and the most common Gram-negative AR bacteria were *A. baumannii*, *K. pneumonia*; the most common Gram-positive AR bacteria were *S. aureus* and *E. faecium*. Although the colistin indicated a highly sensitive antibiotic, resistance of *K. pneumonia* had a median of 21%. Self-antibiotic medication, empirical antibiotic administration, and antibiotics prescribed by general practitioners were the risk factors of high levels of AR during COVID-19. Those prescribing antibiotics should strictly abide by the ASP and guidelines from the WHO and MOH, particularly during pandemics. Healthcare providers and people in the community need more awareness with respect to the proper uses of antibiotics, both during pandemics and in normal situations. Urgent support from policymakers and authorities is needed to issue more restrictions on the uses of antibiotics, more so than in the current situation.

## Figures and Tables

**Table 1:** Summary of characteristics and findings of the 23 studies included.

Author	Country	Year	Study Design	Duration (months)	Settings	Participants	Age	AR Findings	Secondary Infection	Quality
Hassan Mahmoudi	Iran	2020	Cross sectional study	8	Inpatients and outpatients	340 patients	NA	Among COVID-19 patients, Enterobacteriaceae had the highest resistance to cotrimoxazol, piperacillin, ceftazidime, and cefepime.	*Klebsiella*, *S. aureus* (MSSA), *E. coli*, *S. aureus* (MRSA), and Enterobacter species, and *P. aeruginosa*.	Medium 50%
Ehsan Sharifipour	Iran	2020	Retrospective observational study	During the COVID-19 era	Inpatients (ICU)	19 patients	Mean (SD) 67 (± 14.6)	*A. baumannii* isolates showed high-level resistance to all tested antibiotics. Only colistin showed a 52% resistance rate.	*A. baumannii*.	Medium 66%
Jie Li	China	2020	Retrospective electronic medical records reviewed study	2	Inpatients (ICU)	102 patients	Mean (SD) 66.2 (±11.2)	The rate of AR was generally high. Carbapenem-resistant *A. baumannii* (CRAB) and carbapenem-resistant *K. pneumoniae* (CRKP) accounted for 91.7% and 76.6% of AR, respectively. Meticillin resistance was present in 100% of *S. aureus* and coagulase-negative staphylococci. Extended-spectrum beta-lactamase (ESBL) producing *E. coli* was responsible for 75% of AR.	The top three bacteria causing SBIs were *A. baumannii*, *K. pneumoniae*, and *S. maltophilia*.	High 100%
Angela Gomez-Simmonds	United States (New York)	2020	Retrospective study	3	Inpatients (ICU)	13 patients	Median age 67 years, IQR (50–72)	Most of (18/20) the isolates showed high-level meropenem resistance. One patient with *K. pneumoniae* VAP developed ceftazidime/avibactam treatment failure attributable to the development of resistance.	*K. pneumoniae* and 4 *Enterobacter cloacae* complex isolates.	High 100%
Ling Sang	China	2020	Retrospective medical records review study	3	Inpatients (ICU)	190 patients	Mean (SD) 62.68 (±13.3)	The rates of MDR bacteria and CRE were unexpectedly high (*K. pneumonia* = 94.5%, *A. baumannii* = 98.3%, and *Pseudomonas* spp. = 92.5%).	*K. pneumoniae*, *A. baumannii*, *S. maltophilia*, *C. albicans*, and *Pseudomonas* spp.	Medium 50%
Naveenraj Palanisamy	India	2021	Retrospective observational study	5	Inpatients (ICU)	750 patients	Median (IQR) 65 years (54–70)	Out of 64 patients, 57.8% patients had MDRO. The incidence of carbapenem-resistant Gram-negative bacteria was 47.2% (25/53).	*A. baumannii*, followed by *K. pneumonia*.	High 87%
Aleksa Despotovic	Serbia	2021	Retrospective study	12	Inpatients (ICU)	611 patients	Mean (SD) 66.2 (±13.6)	The majority of tested antimicrobials demonstrated high resistant rates, above 80%. Additionally, resistance was significantly higher for imipenem, meropenem, and ciprofloxacin compared with the pre-COVID-19 era.	In COVID-19 patients, Acinetobacter spp. was the dominant cause of HAIs and more frequently isolated than in non-COVID-19 patients.	High 100%
Takwa E. Meawed	Egypt	2021	Cross-sectional study	6	Inpatients (ICU)	197 patients	Range: from 40 to 83 years	The most frequently isolated bacteria were (PDR) *K. pneumoniae*, followed by (MDR) *A. baumannii*.	PDR were *K. pneumoniae*, followed by MDR *A. baumannii*.	High 83%
Basit Zeshan	Pakistan	2021	Retrospective follow-up study	3	Inpatients (ICU)	856 patients	Classified age group. Over 61 was the largest group.	*E. coli* was mostly resistant to ciprofloxacin and ampicillin. *K. pneumoniae* was mostly resistant to ampicillin and amoxycillin.	*E. coli* and *K. pneumonia*.	Medium 66%
Paola Caruso	Italy	2021	Retrospective study	22	Inpatients and outpatients	255 patients	Median (IQR), 65.0 (58.0, 74.0)	Compared with the 2019 group, the 2020 group had a significantly higher prevalence of AR. The prevalence of *S. aureus* resistance to oxacillin and the *C. striatum* resistance to both vancomycin and linezolid was significantly higher in 2020. Regarding the resistance among Gram-negative bacteria, the 2020 group showed a significantly higher rate of resistance to carbapenems, colistin, and third- and fourth-generation cephalosporins.	The most frequent Gram-positive pathogen isolated in both 2019 and 2020 was *S. aureus*, whereas, among Gram-negative bacteria, *P. aeruginosa* was detected more frequently in both cohorts.	High 87%
Vikas Saini	India	2021	Retrospective review study		Inpatients (ICU)	7309 samples pre-pandemic and (4968) samples during the pandemic phase in 2020	Classified age group; however, above 18 Y was significant.	Compared with the pre-COVID-19 era, during COVID-19, bacterial isolates indicated up to 40% of AMR.	Common bacteria during the COVID-19 era included *A. baumannii* and *E. coli*.	High 83%
Eustachius Hagni Wardoyo	Indonesia	2021	Retrospective study	13	Inpatients and outpatients	148 isolates in group A and 62 isolates in group B	NA	An increase in susceptibility was observed in 10/16 antibiotics, where ofloxacin, aztreonam, and fosfomycin were significant. A significant decrease in susceptibility to piperacillin, amoxicillin, cefadroxil, and ampicillin was seen.	The study focuses on *E. coli*.	Medium 66%
Mustafa Karataş	Turkey	2021	Retrospective comparative study	3	Inpatients and outpatients	3532 patients	Median 52 (IQR) (0–99)	The rate of ESBL producing Enterobacterales MDR bacteria pre-COVID-19 was similar to the rate during COVID.	The most common strains pre-COVID-19 and during COVID-19 were the same, as follows: *E. coli*, *K. pneumoniae*, and *P. aeruginosa*.	High 83%
Chiara Temperoni	Italy	2021	Retrospective observational study	3	Inpatients (ICU)	89 patients	Median 67.1 years	Among Gram-negative and Gram-positive bacteria isolates, MDR was 55.2% and 37.2%, respectively.	The most common Gram-negative bacteria were *E. coli*, *A. baumannii* and *K. pneumoniae*. The most common Gram-positive bacteria were *S. aureus* and *E. faecalis*.	Medium 66%
Abdulrahman S. Bazaid	Saudi Arabia	2022	Retrospective study	8	Inpatients and outpatients	108 patients	Classified age group Half of the study cohort was aged 56 years or over	Overall, the AR rate was higher among ICU patients compared with non-ICU patients. In total, 56% of ICU patients infected by *A. baumannii* and *K. pneumoniae* presented with full resistance to all examined antibiotics except colistin. In non-ICU patients, *E. coli* was highly resistant to piperacillin/tazobactam and trimetho-prim/sulfamethoxazole.	The most prevalent bacteria among ICU patients were *A. baumannii* and K. pneumoniae. In non-ICU patients, *E. coli* and P. aeruginosa were predominant organisms.	Medium 66%
Samaneh Pourajam	Iran	2022	Retrospective study	6	Inpatients (ICU)	553 patients	Median (IQR) 69.4 (21–95) years	Most patients had XDR. The prevalence of carbapenem-resistant Gram-negative bacilli in COVID-19 patients was high.	*K. pneumonia* and *A. baumannii*	High 83%
Alireza Nikzad Jamnani	Iran	2022	Retrospective cohort study	7	Inpatients (ICU)	38 patients	Classified age group. >of 70 years represented the majority.	Acinetobacter spp. had 100% resistance to amikacin, gentamycin, imipenem, and cefxime. Additionally, *Klebsiella* spp. had 100% resistance to amikacin, cotrimaazol, cefxime, ceftazidime, gentamycin, and ciprofloxacin. The resistance of *E. coli* to cefxime and cotrimaazol in the corona group was 100%. Among the non-corona group, Acinetobacter spp. and *Klebsiella* spp. were resistant to almost all tested antibiotics except colistin.	*Acinetobacter* spp. were the most common bacteria.	Medium 66%
Marina Gysin	Switzerland	2021	Prospective observational study	2	Inpatients (ICU)	168 isolates	NA	High resistance was found in P. aeruginosa for piperacillin/tazobactam, cefepime, ceftazidime, and meropenem. Low levels of resistance were found in Enterobacterales for piperacillin/tazobactam, ceftriaxone, and ceftazidime.	*P. aeruginosa*, *Enterobacter cloacae*, and *Klebsiella*.	Medium 66%
Michalis Polemis	Greece	2021	Retrospective observational study	36	Inpatients and outpatients	17,837 isolates	NA	Significant differences were found in the slope of non-susceptibility trends of 1- *A. baumannii* to amikacin, tigecycline, and colistin; 2- *K. pneumoniae* to meropenem and tigecycline; 3- *P. aeruginosa* to imipenem, meropenem, and levofloxacin. Additionally, significant differences were found in the slope of non-susceptibility trends of *S. aureus* to oxacillin and of *E. faecium* to glycopeptides.	The most common bacteria were A. baumannii, K. pneumoniae, P. aeruginosa, and E. coli.	Medium 66%
Yasemin Genç Bahçe	Turkey	2022	Retrospective observational study	22	Inpatients (ICU)	119 isolates before COVID-19; 87 isolates afterwards.	Mean (SD) 71.36 (± 14.93)	AR rates in *A. baumannii* strains increased following the pandemic, except for tigecycline. High AR was observed after the pandemic *for K. pneumoniae*; however, these increases were not statistically significant. Except for imipenem, antibiotic resistance rates in *P. aeruginosa* strains increased following the pandemic.	*A. baumannii*, *K. pneumoniae*, *P. aeruginosa*, and *E. faecium* were the most common in the pandemic time.	High 83%
Khaled Abdulrahman Aldhwaihi	Saudi Arabia	2021	Retrospective study	7	Inpatients and outpatients	286 isolates	NA	AR rates were congruent before and during COVID-19 pandemic.	*A. baumannii*, *K. pneumonia*, *E. Coli*.	Medium 50%
Sushma Yadav Boorgula	India	2022	Retrospective study	2	Inpatients	122 patients	Median (IQR) 58 (51.67)	Bacterial resistance to Carbapenem had an 6% increase among tested isolates.	*K. pneumoniae* followed by *A. baumannii*.	High 100%
Surbhi Khurana	India	2021	Prospective study	3	Inpatients (ICU)	151 patients	Mean (SD) 46.01 ± 19.03	The hitherto observed resistances were as follows: amoxicillin/clavulanic acid = 84%, levofloxacin = 83%, ciprofloxacin = 79%, piperacillin/tazobactam = 77%, and trimethoprim/sulfamethoxazole = 75%. Generally, resistance to third-generation cephalosporins and carbapenems was (64%– 69%). Notably, all isolates were found to be sensitive to colistin.	*K. pneumonia*, *A. baumannii*, *E. coli*, and *P. aeruginosa*.	High 100

NA: Not available. High: score ≥ 70%. Medium: score 50–69%. Low: score <50%.

**Table 2:** Gram-negative bacteria resistant to tested antibiotics during COVID-19.

Antibiotic	*A. baumannii* Median (IQR)	*E. coli* Median (IQR)	*K. pneumonia* Median (IQR)	*P. aeruginosa* Median (IQR)
Amoxicillin clavulanate	-	85.5 (49–92.75)	81.8 (79.3–83.75)	-
Amikacin	84.6 (56.3–92.95)	6 (0–43,35)	69.85 (58.7–80.12)	25 (12–28)
Ampicillin	-	87.5 (85.25–93.75)	100 (90.5–100)	-
Aztreonam	-	-	84.7 (67.27–88.87)	-
Cefazolin	-	-	93 (78–95.5)	-
Cefuroxime	-	65.5 (55.75–77.62)	88.9 (79.6–91.42)	-
Cefepime	94.4 (93–100)	0 (0–25)	81.15 (71.7–87.25)	14.3 (12.5–47.8)
Ceftazidime	91.2 (50–100)	18.75 (0–41.87)	93.5 (83.7–97.9)	40 (23–41.7)
Cefoperazone sulbactam	-	-	76.2 (73.8–77.9)	-
Ceftriaxone	76.2 (54.75–95.55)	73 (49.25–93.75)	84 (77.55–93.4)	75 (43.75–87.5)
Colistin	2.5 (0–19.62)	0 (0–7.15)	21.1 (12.42–69.82)	4 (0–12.25)
Gentamicin	95.7 (74.2–97.1)	40 (19–47)	57.1 (33.45–86.6)	25 (19.75–58.75)
Levofloxacin	97.05 (91.92–100)	75 (56.85–87.5)	80.8 (78.55–90.85)	43.5 (28.6–80)
Meropenem	92.1 (64.02–95.65)	2.5 (0–26.17)	71.25 (55.37–77.37)	38 (18.37–42.17)
Imipenem	92.1 (80.65–95.72)	10 (4–26)	65.7 (19.25–72.87)	42.9 (19.75–52.9)
Ertapenem	-	-	71.4 (55.55–75.05)	-
Ciprofloxacin	91.2 (65–100)	71 (28.2–76)	87.8 (55.1–92.95)	50 (32.3–62.5)
Trimethoprim/sulfamethoxazole	50 (46.8–84.2)	50 (40–80)	73.5 (32–74)	-
Tigecycline	9.5 (8.8–33.3)	0 (0–22.5)	31.4 (1.7–44)	-
Piperacillin/tazobactam	93.7 (66.9–100)	23 (12–37.8)	77.7 (57.1–79.27)	11.25 (9.25–13.85)
Nitrofurantion	-	-	51.8 (38.5–60.6)	-

**Table 3:** Gram-positive bacteria resistant to tested antibiotics during COVID-19.

	*S. aureus* Median (IQR)	*E. faecium* Median (IQR)
Oxacillin	48.5 (25.5–63.75)	-
Ampicillin	-	81.8 (52.4–90.9)
Erythromycin	-	90.9 (78.45–95.45)
Clindamycin	33.3 (16.65–50.9)	-
Ciprofloxacin	-	81.8 (77–100)
Vancomycin	-	11 (0–18.1)
Tetracycline	-	66 (60.25–83)

## Data Availability

Data were obtained from PubMed, MEDLINE, EMBASE, Scopus, Cochrane, Web of Science, and CINAHL, and are available on the websites.
